# A one-year prospective study of the safety, tolerability and pharmacokinetics of the highest available dose of paliperidone palmitate in patients with schizophrenia

**DOI:** 10.1186/1471-244X-12-26

**Published:** 2012-03-28

**Authors:** Danielle Coppola, Yanning Liu, Srihari Gopal, Bart Remmerie, Mahesh N Samtani, David W Hough, Isaac Nuamah, Ahmad Sulaiman, Gahan Pandina

**Affiliations:** 1 Janssen Research & Development, LLC, Raritan, New Jersey, USA; 2 Janssen Research & Development, Division of Janssen Pharmaceutica N.V., Beerse, Belgium; 3 Department of Psychological Medicine, Faculty of Medicine, University of Malaya, 50603 Kuala Lumpur, Malaysia

**Keywords:** Antipsychotics, Paliperidone palmitate, Pharmacokinetics, Schizophrenia, Safety, Long-term therapy

## Abstract

**Background:**

There are no previous reports of paliperidone palmitate's (PP) long term tolerability or pharmacokinetics of the highest dose in patients with schizophrenia. This study evaluates safety and tolerability, as well as pharmacokinetics, of the highest marketed dose of PP (150 mg eq. [234 mg]) in stable patients with schizophrenia over a 1-year period.

**Methods:**

In this 1-year prospective study, eligible patients (aged 18-65 years; Positive and Negative Syndrome Scale's total score ≤ 70) received an initial deltoid injection of PP 150 mg eq. The second injection one week later and subsequent once-monthly injections were deltoid or gluteal. All injections were to be PP 150 mg eq. Patients willing to participate in intensive pharmacokinetic sampling were classified as Treatment A. Patients unwilling to undergo intensive pharmacokinetic sampling or unable to tolerate the 150 mg eq. dose (consequently receiving flexible doses of 50, 100 or 150 mg eq.) were classified as Treatment B.

**Results:**

Of the 212 patients (safety analysis set), 73% were men; 45% white; 20% black; 34% Asians; mean (SD) age 41 (10.2) years, and mean (SD) baseline Positive and Negative Syndrome Scale total score 54.9 (9.03). A total of 53% (n = 113) patients completed the study and 104 received PP 150 mg eq. throughout. Mean (SD) mode dose of PP was 144.8 (19.58) mg eq. The dosing initiation regimen resulted in rapidly achieved and maintained therapeutic paliperidone levels over the study (average concentrations during the dosing interval were 34.7, 40.0, and 47.8 ng/mL after the 2nd, 8th, and 14th injection respectively). Most frequent (≥ 10%) treatment-emergent adverse events were nasopharyngitis (n = 37), insomnia (n = 32), injection-site pain (n = 32), headache (n = 28), and tachycardia (n = 27). Akathisia (n = 19) and tremor (n = 11) were the most common extrapyramidal adverse events. 33 patients had an SAE and 27 discontinued due to treatment-emergent adverse events. No deaths were reported. Mean (SD) weight change from baseline was 2.5 (5.41) kg at endpoint. Patients' psychoses remained stable.

**Conclusions:**

Safety results after one-year therapy with the highest available dose of once-monthly paliperidone palmitate were consistent with results from previous studies, with no new concerns noted. Plasma concentrations were within the expected range.

**Trial registration no:**

ClinicalTrials.gov: NCT01150448

## Background

Schizophrenia is a serious and chronic mental disorder requiring continuous and long-term therapy with antipsychotics [1]. Treatment non-adherence to daily oral antipsychotics and the associated risk of symptom relapse or recurrence are some of the global challenges prevalent among patients with schizophrenia [2,3]. As compared with the oral formulations, the long-acting injectable (LAI) formulations of antipsychotic drugs have the advantage of a sustained release profile providing therapeutic plasma concentration for several weeks [4], thereby eliminating the need for daily oral medication [5-7]. Thus, LAIs ensure continuous drug exposure, mitigating the risk of nonadherence associated with oral agents, and can provide symptom control with lower overall plasma concentrations [1,8].

Paliperidone palmitate (PP) is a once-monthly injectable atypical antipsychotic recently approved in the United States for the acute and maintenance treatment of schizophrenia in adults [9]. Treatment with PP does not require oral supplementation during initiation, as its pharmacokinetic (PK) profile allows both a rapid achievement of therapeutic plasma levels of paliperidone as well as a gradual and continuous release of the drug over the dosing interval [10]. The approved recommended initiation regimen [9] with deltoid injections of a high dose of PP (150 milligram equivalent [mg eq.]. on day 1 and 100 mg eq. on day 8) allows rapid attainment of therapeutic concentrations [11,12].

In this phase-1 study, a higher than recommended 2^nd ^dose in the initiation regimen of PP (150 mg eq. on day 1 and 150 mg eq. on day 8) and the highest recommended maintenance dose were used to evaluate the long-term (up to 1 year) safety and tolerability of the highest available dose of PP 150 mg eq. and to evaluate the PK data at steady state, which is expected to occur after 6-8 injections of PP 150 mg eq.

## Methods

### Patients

Consenting men and women aged, 18 to 65 years (inclusive) who were diagnosed with schizophrenia per Diagnostic and Statistical Manual of Mental Disorders, Fourth Edition (DSM-IV) at least one year before screening and with a PANSS total score of ≤ 70 at screening were enrolled. Other important inclusion criteria included: Body Mass Index (BMI) of ≥ 17.0 kg/m^2 ^at screening, and ability to provide informed consent, including refusal or consent for optional pharmacogenomic research.

Main exclusion criteria included: primary active DSM-IV Axis I diagnosis other than schizophrenia, involuntary commitment to psychiatric hospitalization, previous treatment with injectable antipsychotic formulations other than PP or risperidone-LAI) within 1 injection interval on day 1, previous PP injection within 10 months or risperidone-LAI injection within 100 days before day 1, at significant risk of suicidal or violent behavior, history of neuroleptic malignant syndrome or tardive dyskinesia (TD), hypersensitivity to risperidone, paliperidone or their excipients, history of significant or unstable systemic disease, or history or presence of circumstances that would increase the risk of torsade de pointes or sudden death associated with the use of drugs that prolong QT interval.

### Study design

This was a phase 1 open-label, long-term, multiple-dose, and multicenter study conducted from September 2007 to June 2009 at 30 centers in 10 countries (Belgium, Croatia, Spain, the Republic of Korea, Malaysia, Poland, Slovakia, Thailand, Taiwan, and the United States) in accordance with the ethical principles that have their origin in the Declaration of Helsinki, and that was consistent with Good Clinical Practices and applicable regulatory requirements. The Independent Ethics Committee or Institutional Review Board at each study site approved the protocol and all patients provided written informed consent prior to entering the study.

**Figure 1:**
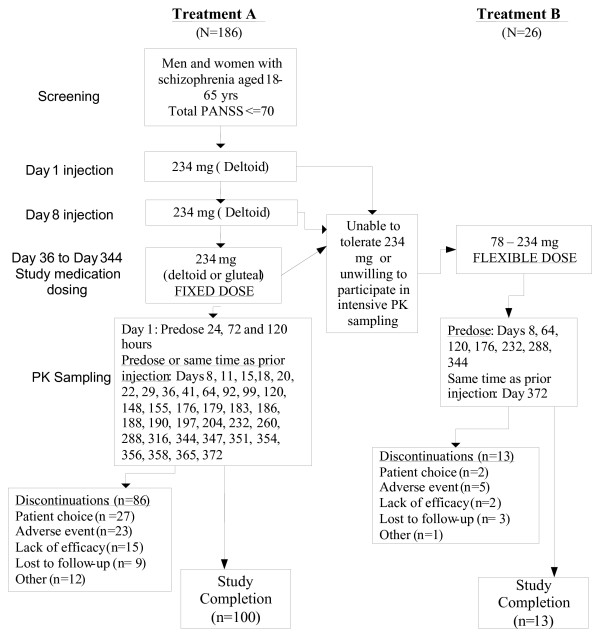
**Study Design**. PK - pharmacokinetic.

The study consisted of a screening and washout phase of up to 21 days (including oral tolerability testing with paliperidone extended release [ER] 6 mg/day for 4 to 6 consecutive days for patients without documented previous exposure to risperidone or paliperidone) and a 53-week open-label treatment phase (approximately 56 weeks in total) (Figure 1). All injections were planned to be PP 150 mg eq. Patients on PP 150 mg eq. willing to participate in intensive PK sampling formed the primary analysis group (Treatment A). Patients who declined to participate in Treatment A, either due to the intensive PK sampling or if they could not tolerate the 150 mg eq. dose of PP, were still allowed to participate in Treatment B with less frequent PK sampling. All patients began the study entering into Treatment A, and were allowed to switch to Treatment B (dose range 50-150 mg eq.) after the first injection, at the discretion of the investigator.

During the study, previous oral or injectable antipsychotics (except risperidone, paliperidone ER or clozapine) were to be stopped 3 days before the start of the study. Clozapine was not allowed within 6 weeks before day 1 and risperidone or paliperidone ER were discontinued at least 5 days before the first injection. Antiparkinsonism drugs were allowed only if necessary, and were otherwise tapered to discontinuation 1 day before the screening or washout phase.

All patients received an initial dose of PP 150 mg eq. in the deltoid muscle (day 1) and the subsequent second injection (day 8) in the deltoid (Treatment A), or in either the deltoid or gluteal muscle (Treatment B). In both groups, the 12 subsequent injections were administered every 4 weeks in either the deltoid or gluteal muscle alternating between sides (left/right) (Figure 1). Psychiatric and safety assessments were performed periodically throughout the study.

#### Study medication

Paliperidone palmitate doses can be expressed both in terms of mg eq. of the pharmacologically active fraction, paliperidone, and in mg of PP. Thus, the doses expressed as PP 50, 100, or 150 mg eq. equate to PP 78, 156, and 234 mg, respectively. Paliperidone palmitate was supplied as injectable suspensions (50,100, 150 mg eq.) in prefilled syringes with 22 and 23 gauge needles. Needle gauge was based on intramuscular injection site (deltoid or gluteal) and patient body weight.

### Study assessments

#### Pharmacokinetic assessments

Throughout the study, 4 mL of venous blood samples were taken at all visits for Treatment A and at selected visits for Treatment B (Figure 1) to determine plasma paliperidone concentration.

Samples were collected before study drug administration (if scheduled). On days when 12-lead ECGs were recorded, the PK blood samples were collected as soon as possible after the 12-lead ECG. Plasma concentrations of paliperidone were determined in 2 laboratories (Bioanalytical Department, Janssen Research & Development, LLC, Beerse, Belgium and Frontage Laboratories Co., Ltd., Shanghai, China). A validated liquid chromatography coupled to tandem mass spectrometry (LC-MS/MS) methods with a target limit of quantification (LOQ) of 0.1 ng/mL was used. Before sample analysis, a cross-validation of study samples previously analyzed by Frontage Laboratories was performed. Pharmacokinetic analysis and descriptive statistics were performed using PKAA version 2.1b (developed by Intrasphere Technologies, London, U.K); and using WinNonLin 5.2.1 (Pharsight, Mountain View, California, U.S.).

The plasma PK parameters estimated for each patient in Treatment A who had intensive PK sampling included: i) plasma concentration measured immediately before the i.m. injections (C_predose_); ii) observed maximum plasma concentration (C_max_) and minimum plasma concentration (C_min_), time to reach the maximum plasma concentration (t_max_), and area under the plasma concentration-time curve within the interval of 28 days (AUC_τ_) after the 2^nd^, 8^th^, and 14^th ^injections; iii) average plasma concentration (C_avg_), calculated as the AUC_τ _after the 2^nd^, 8^th ^and 14^th ^injections divided by the dosing interval; iv) fluctuation Index (FI): percentage fluctuation (variation between peak and trough at steady-state), calculated as 100*[(C_max_-C_min_)/C_avg_].

#### Population-PK model

The historical model [11] was examined for its predictive ability and its performance was evaluated on the current data set that was not used in model building exercise. Population predictions for all concentrations in the evaluation data set were obtained using the dosing and necessary covariate information. Prediction errors were computed that provide a measure of bias and precision by assessing the differences between the measured and population mean predicted concentrations. The prediction error percents (PE%) were computed for each concentration value using the equation: PE%_ij _= (DV_ij_- PRED_ij_)/PRED_ij_* 100, where, *PE%_ij _*is the percent prediction error between the *i^th ^*value of the dependent variable in the *j^th ^*subject; and the population prediction of the *i^th ^*observation in the *j^th ^*subject; *DV_ij _*is the value of the *i^th ^*observation (i.e. the dependent variable) in the *j^th ^*subject; and PRED_ij _is the population prediction of the *i^th ^*observation in the *j^th ^*subject.

The absolute prediction error percents (|PE|%) was computed as the absolute value of the PE%. Summary statistics of PE% and |PE|% were calculated to assess bias and precision of the model-predicted concentrations relative to the observed concentrations. The distribution of PE% was evaluated as a measure of bias and the distribution of |PE|% was utilized as a measure of precision in the predicted values. The model was considered acceptable, both accurate and precise, (i.e., externally validated) if the median PE% and the median |PE|% were ≤ |15|% and 30%, respectively [11,13-15].

In addition, the model was subjected to model verification using a visual predictive check [11]. Finally, the population PK model was also used to evaluate the effect of ethnicity and BMI on the PK of PP.

#### Safety assessments

Safety assessments included evaluation of treatment-emergent adverse events (TEAEs), extrapyramidal symptoms (EPS) rating scores based upon the Barnes Akathisia Rating Scale (BARS) [16], Simpson-Angus Rating Scale [17] (SAS), and Abnormal Involuntary Movement Scale (AIMS) [18], changes in weight, electrocardiograms, vital signs, clinical laboratory data, and subjective assessment of injection-site pain.

#### Psychiatric assessments

Psychiatric evaluations included PANSS [19], PSP (Personal and Social Performance scale) [20], and CGI-S (Clinical Global Impression-Severity scale) [18], which were also periodically evaluated by qualified raters to monitor patient symptoms and to adjust concomitant psychiatric medications if required.

### Statistical methods

#### Sample size determination

Using an estimated intrapatient coefficient of variation of 40% for AUC_τ _and C_max _of paliperidone after PP injection, and a 5% significance level, a sample size of 40 patients from Treatment A was considered sufficient for the point estimate of the relative bioavailability after the 8^th ^i.m. injection of PP 150 mg eq. (the highest available dose) compared to the 2^nd ^i.m. injection of PP 150 mg eq. to fall within 86.0% and 116.3% of the true value, with 90% confidence.

A minimum of 100 patients exposed for 1 year was considered to be an adequate group size and time period to assess safety and tolerability. Assuming a discontinuation rate of 50%, it was estimated that a total of 200 patients (from either Treatment A or Treatment B) would need to be enrolled so that at least 100 patients would complete the study.

#### Pharmacokinetic analysis

To assess the attainment of apparent steady-state after a loading dose and to estimate the relative bioavailability of paliperidone after the 8^th ^injection compared to the 2^nd ^injection, different PK analyses and explorations were conducted, requiring different exclusion strategies. The PK data were analyzed using a mixed-effect analysis of variance model that included the 2^nd^, 8^th ^or 14^th ^injections as a fixed effect and patient as a random effect.

Using the estimated least-squares means and intrasubject variance, the point estimate and 90% confidence intervals for the difference in means on a log scale between the 14^th ^or 8^th ^and 2^nd ^i.m. injections were constructed. The limits of the confidence intervals were retransformed using antilogarithms to obtain 90% confidence intervals for the ratios of the mean AUC_τ _and C_max _after the 14^th ^or 8^th ^i.m. injections of PP 150 mg eq. versus the 2^nd ^i.m. injection of PP 150 mg eq. As per study design, no formal statistical comparison of PK results of patients in Treatment B was performed.

#### Safety analysis

All patients who received at least 1 dose of the study drug were included in the safety analyses. Only descriptive statistics were performed.

#### Psychiatric symptom assessment analysis

All patients who received at least 1 dose of the study drug and who had at least 1 postbaseline psychiatric evaluation were included in the statistical analyses of the psychiatric evaluations. The PANSS, CGI-S, and PSP scores were summarized descriptively.

#### Exploratory analysis

Exploratory analysis of the safety data was performed to compare the safety profile of PP in patients who completed the trial while receiving 150 mg eq. throughout and those who did not complete the study at 150 mg eq. dose of PP (discontinued the dose of 150 mg eq. of PP or received flexible dose [50-150 mg eq.] at any time).

## Results

### Patient disposition and population

Of the 212 enrolled patients, 113 (53%) completed the study (Figure. 1). Overall, most of the patients were between 26 to 50 years, most were men and were white. The mean (SD) age was 40.7 (10.17) years (range: 19 to 65 years) (Table 1).

**Table 1:** Demographic and baseline characteristics (safety analysis set)

	Treatment A	Treatment B	
	**PP 150 mg eq**.	**PP 50-150 mg eq**.	**Total**

	**(N = 186)**	**(N = 26)**	**(N = 212)**

**Age (years)**			

Mean (SD)	41.4 (10.24)	35.7 (8.17)	40.7 (10.17)

**Sex, n (%)**			

Men	138 (74)	16 (62)	154 (73)

Women	48 (26)	10 (38)	58 (27)

**Race, n (%)**			

White	88 (47)	8 (31)	96 (45)

Black	36 (19)	6 (23)	42 (20)

Asian	60 (32)	12 (46)	72 (34)

Other^a^	2 (1)	0	2 (< 1)

**Ethnicity, n (%)**			

Not Hispanic or Latino	184 (99)	24 (92)	208 (98)

Hispanic or Latino	2 (1)	2 (8)	4 (2)

**Weight (kg)**			

Mean (SD)	78.1 (18.55)	73.5 (16.45)	77.5 (18.33)

**Height (cm)**			

Mean (SD)	170.6 (10.24)	171.0 (9.97)	170.6 (10.18)

**Baseline body mass index (kg/m^2^**)			

Category, n (%)			

Normal < 25	70 (38)	14 (54)	84 (40)

Overweight 25- < 30	66 (35)	7 (27)	73 (34)

Obese ≥ 30	50 (27)	5 (19)	55 (26)

Mean (SD)	26.71 (5.55)	24.91 (4.16)	26.49 (5.42)

**Psychiatric Evaluations^b^**			

PANSS total scores, Mean (SD)	55.5 (8.87)	51.5 (9.86)	55.0 (9.08)

PSP, Mean (SD)	67.3 (10.41)	68.0 (10.01)	67.4 (10.33)

CGI-S, Median (Range)	3.0 (2;6)	3.0 (2;5)	3.0 (2;6)

^a ^includes native Hawaiian or other Pacific islander; ^b ^For PANSS and PSP: n = 178 (Treatment A), n = 26 (Treatment B), and n = 204 (Total); For CGI-S: n = 182 (Treatment A), n = 26 (Treatment B), and n = 208 (Total); PANSS- Positive and Negative Syndrome scale; PSP- Personal and Social Performance Scale; CGI-S- Clinical Global Impression Severity Score; PP- Paliperidone palmitate, BMI- Body Mass Index

All patients in the safety analysis set (N = 212) were initially assigned to Treatment A (to receive fixed doses of 150 mg eq. PP throughout and participate in intensive PK sampling). Out of these, 12% of patients (n = 26) switched to Treatment B, which allowed them to either receive continued treatment with PP 150 mg eq. or to choose a flexible dose option (50-150 mg eq.). All patients in Treatment B had less frequent PK sampling than those in Treatment A. Out of the 113 completers, 104 (Treatment A: n = 100; Treatment B: n = 4) received the 150 mg eq. dose regimen for the entire study. The major reasons for withdrawal were patient choice (14%) and adverse events (13%). Nearly 50% of all patients were exposed to PP for at least 344 days. Total mean duration of exposure was 250 days (Treatment A: 248 days, Treatment B: 264 days). The overall mean (SD) mode dose (mean of the most frequently used dose) of PP was 144.8 (19.58) mg eq. (Treatment A: 150 mg eq.; Treatment B: 109.6 mg eq.).

In this study, patients in Treatment A formed the primary analysis group, and all interpretations are presented for this group. Interpretation of safety results for Treatment B is limited since only a few patients in this group (n = 7 out of 26; with 4 completing) continued to receive PP 150 mg eq. dose, and the reason for dose adjustments in this group were not systematically documented. Overall, 16 patients had dose adjustments due to a TEAE.

The effect of repeated deltoid administration could be assessed in 46 patients (Treatment A: n = 44; Treatment B: n = 2) who received 14 consecutive deltoid injections of PP. Frequently used concomitant benzodiazepines during the study included lorazepam (38%), diazepam (16%) and clonazepam (10%). Zolpidem (29%) was the most frequently used non-benzodiazepine drug.

### Pharmacokinetic results

**Figure 2:**
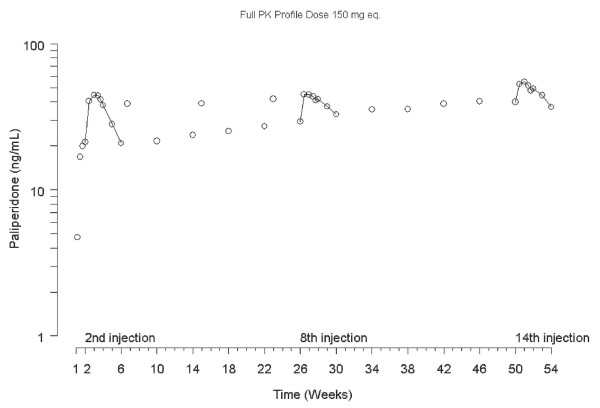
**Median plasma concentration-time profiles of paliperidone (data from 212 patients)**. Time-concentration profiles were based on data from 212 patients.

**Figure 3:**
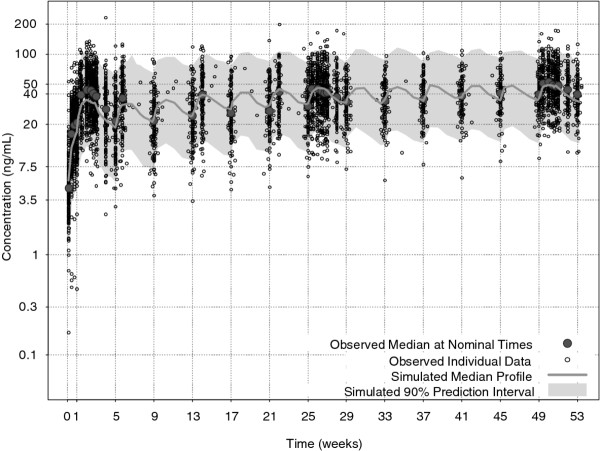
**Visual predictive check comparing results from the population PK simulation to the actual observed plasma concentrations**. PK-pharmacokinetic. Data included is of 192 patients who received 150 mg eq. paliperidone palmitate till the time of their discontinuation.

Paliperidone exposure approached the exposure at steady state from the second injection onwards, with the steady-state levels reached after about 8 to 9 months (Figure 2). The median average plasma concentration (C_avg_) of paliperidone continued to increase over the injection period (Table 2). The median C_max _of paliperidone was comparable after the 2^nd ^and 8^th ^injections and slightly higher after the 14^th ^injection of PP (Table 2). The median fluctuation index gradually decreased over the injection period. The AUC_τ _was slightly increased after the 8^th ^injection compared with the 2^nd ^injection. Mean exposure to PP increased and maximum plasma concentrations were higher after the 8^th ^and 14^th ^injections compared with the 2^nd ^injection (Table 3). Overall, the results indicated that within one week after the first dose, the majority of patients had plasma concentrations above 7.5 ng/mL (Figure 3), the minimum required plasma paliperidone concentration associated with a central D2-receptor occupancy of approximately 60% (Karlsson et al., 2006) that is generally considered the threshold for antipsychotic efficacy. A median plasma paliperidone concentration of 7.5 ng/mL was reached between the 2^nd ^and 3^rd ^PK visits (day 2 and day 4).

**Table 2:** Median pharmacokinetic parameters after intramuscular injections of paliperidone palmitate (150 mg eq.)

	2^nd ^injection		8^th^ injection		14^th^ injection	
**PK parameter**	**N**	**Median ** **(min-max)**	**N**	**Median ** **(min-max)**	**N**	**Median** ** (min-max)**

C_predose_, ng/mL	200	21.3 (0.46-81.2)	119	28.4 (6.03-92.6)	105	39.9 (9.17-134)

C_min_, ng/mL	189	17.0 (0.46-46.7)	114	27.0 (6.03-92.6)	105	35.1 (9.17-120)

C_max_, ng/mL	189	50.5 (11.5-232)	114	50.5 (10.7-172)	105	56.5 (17.6-172)

t_max_, days^a^	189	7.96 (0.00-28.02)	114	8.48 (0.00-30.95)	105	7.00 (0.00-30.00)

AUC_tau_, h*ng/mL	183	23325 (3652-86457)	111	26831 (6335-87393)	105	31970 (9244-96840)

C_avg_, ng/mL	183	34.7 (5.44-129)	114	40.0 (9.42-130)	105	47.8 (13.8-144)

FI, %	183	92.7 (39.8-215)	114	55.0 (15.6-179)	105	41.2 (5.83-111)

^a^t_max _was calculated in hours and recalculated to days here. FI = fluctuation index

**Table 3:** Plasma pharmacokinetic parameters of paliperidone following injections of paliperidone palmitate (150 mg eq.) (pharmacokinetic analysis set)

			Geometric	Ratio	Confidence Interval
**PK parameter**	**Injection**	**N**	**LSM**	**(Test/Reference)^a^**	**(90%)**

AUC_τ_, ng.h/mL	2nd injection	96	21924.25		

	8th injection	96	26638.65	121.50	(114.42;129.03)

	14th injection	96	31522.41	143.78	(135.39;152.68)

C_max_, ng/mL	2nd injection	99	46.74		

	8th injection	99	49.42	105.75	(98.82;113.16)

	14th injection	99	57.74	123.55	(115.46;132.21)

Note: Data analyzed on log-scale but transformed back to the original scale					

LSM = Least-Squares Means					

^a ^Test refers to 8th/14th injection and Reference refers to 2nd injection

The overlay plots comparing results from the population PK simulation to the actual observed plasma concentrations showed considerable similarity and the majority of the observations were within the 90% prediction interval, confirming agreement between anticipated temporal PK profile and observed PK data (Figure 2). Prediction errors were well within cut-off values and statistical distributions supported the suitability of the model (Table 4). Although the model was developed on PK data obtained from 25 weeks treatment after 7 injections of PP [11], the model also adequately predicted the PK of paliperidone for 53 weeks treatment after 14 injections (Figure 3).

**Table 4:** Tabular results for the external validation: distribution for PE% and |PE|% to assess bias and precision respectively^a^

	N^b^	Observed Median	Median cut-off to pass validation	Observed 25^th ^percentile	Observed 75^th ^percentile
Prediction error percents (PE%)	5382	0.72	± 15	-9.37	10.47

Absolute prediction error percents (|PE|%)	5382	9.96	30	4.55	17.65

^a ^The prediction error percents (PE%) were computed for each concentration value using the equation below and the absolute prediction error percents (|PE|%) was computed as the absolute value of the PE%. PE%_ij _= (DV_ij_- PRED_ij_)/PRED_ij_* 100, where, *PE%_ij _*is the percent prediction error between the *i^th ^*value of the dependent variable in the *j^th ^*subject; and the population prediction of the *i^th ^*observation in the *j^th ^*subject; *DV_ij _*is the value of the *i^th ^*observation (i.e. the dependent variable) in the *j^th ^*subject; and PRED_ij _is the population prediction of the *i^th ^*observation in the *j^th ^*subject.

^b^N refers to the number of pharmacokinetic samples used in the calculation of the PE% and |PE|% statistics. The 5382 pharmacokinetic samples were derived from 211 patients.

Using the historical population PK model, the time to reach 97% of the maximal median concentration achieved after one year of dosing was calculated. Attainment of 97% steady-state is equivalent to the frequently used criterion of five times the effective half-life. The model predicted that 97% of steady state would be achieved after 38 weeks for multiple deltoid injections of 100-150 mg eq. PP and 42 weeks for multiple gluteal injections of 100-150 mg eq. PP. The observed median plasma concentration profile shows that by week 41, 97% of the maximal median concentration was achieved, indicating that the expectation from the population-PK model has been met.

The observed median plasma concentration-time profiles of paliperidone from Asians and White patients showed that Asians had approximately 4% higher plasma exposure to paliperidone compared with White patients (see Additional file 1), which can be explained by the lower BMI in the Asian population (see Additional file 2). Furthermore, the population PK analysis showed that race does not have an independent effect on PK once the difference in BMI across ethnicities is accounted (see Additional file 3).

### Safety results

#### Treatment-emergent adverse event

**Figure 4:**
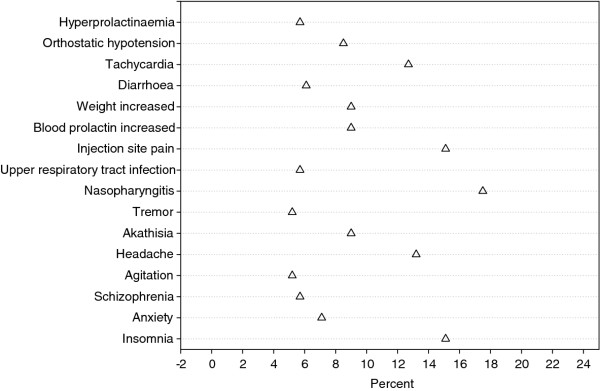
**Treatment-emergent adverse events experienced by at least 5% of patients (Safety Analysis Set)**.

About 184 patients (87%; safety analysis set: n = 212) experienced at least 1 TEAE during the study. The majority of these TEAEs were mild to moderate in severity. The most frequent TEAEs (in ≥ 10% of all patients) were nasopharyngitis (17.5%), insomnia (15%), injection-site pain (15%), tachycardia (13%), and headache (13%) (Figure 4). There were no deaths reported during the study. Overall, 33 patients (15.6%) had serious TEAEs; among which worsening of symptoms of schizophrenia (5%) and psychotic disorder (3%) were most commonly reported. In all, 27 patients discontinued the study due to a TEAE (Treatment A: n = 22; Treatment B: n = 5), mostly due to psychotic disorders (7.5%), and 11 patients discontinued due to a serious TEAE (Treatment A: n = 10; Treatment B: n = 1). Of the 19 patients who had dose adjustments in Treatment B, dose adjustments were mostly due to a TEAE (in 16 of 19 patients). Only dystonia, orthostatic hypotension, asthenia and sluggishness (n = 2 each) were experienced by more than one patient.

There was no clinically meaningful difference in the incidence of serious TEAEs between patients who exclusively received deltoid injections (12.9%) and those who did not receive injections exclusively in the deltoid (15.6%; safety analysis set). No patient discontinued from the study due to an adverse event related to the injection site (e.g. pain).

#### Other safety events of clinical interest

The majority of patients continued in Treatment A (88%). The numbers of patients who received PP 150 mg eq. dose throughout the study in Treatment B (n = 7) were not sufficient to conduct a valid comparison with Treatment A (n = 186).

Overall body weight increases of ≥ 7% (from baseline to endpoint) occurred in 27% (n = 55) of patients (Treatment A: n = 55; Treatment B: n = 10). At endpoint, mean body weight increased by 3.9% and mean (SD) weight increased 2.5 (5.41) kg from baseline. All patients with an adverse event of diabetes mellitus (< 2%; n = 4) were either obese or overweight and had either diabetic or prediabetic range fasting plasma glucose at screening or baseline. One of the 4 patients had a history of impaired glucose tolerance, and 1 had a baseline elevation of hemoglobin A1_C_. There were no other treatment-emergent glucose-related adverse events. There were no clinically relevant mean changes from baseline to any timepoint in lipids.

EPS-related TEAEs occurred in 50 (of 212, safety analysis set) patients (24%) during the study (Treatment A [20%; n = 38/186] Treatment B [46%; n = 12/26]). The majority of these were non-serious, mild to moderate in intensity. Data on preexisting EPS-related events of the patients enrolled were not collected. After treatment initiation, the proportion of EPS-related TEAEs was < 5% (n = 7; 3%) from day 1 to day 8; increased to 13% (n = 28) during subsequent month (from day 9 to day 36), and subsequently remained < 5% per month interval thereafter. Overall, 30% of the enrolled patients (n = 64) had received prior anti-EPS medication and 10% of these patients (n = 21) continued to receive anti-EPS medications in the study. A total of 22% of patients (n = 46) received anti-EPS medication during the study. Five patients required PP dose adjustments due to EPS-related TEAEs. Overall, 4 patients experienced serious EPS-related TEAEs and 4 patients (all from Treatment A) discontinued due to EPS-related TEAEs. EPS-related TEAEs occurring in at least 5% of total patients were akathisia (n = 19) and tremor (n = 11).

Based on mean changes in EPS rating scales, patients were slightly more symptomatic for akathisia, dyskinesia, and parkinsonism at endpoint versus baseline. At endpoint, overall rates were: 1%, akathisia (n = 3; based on BARS clinical rating); 2%, dyskinesia (n = 5; based on AIMS score); and 16%, parkinsonism (n = 34/212; based on SAS global score) (Treatment A [16%; n = 29/186]; Treatment B [19%; n = 5/26]).

**Figure 5:**
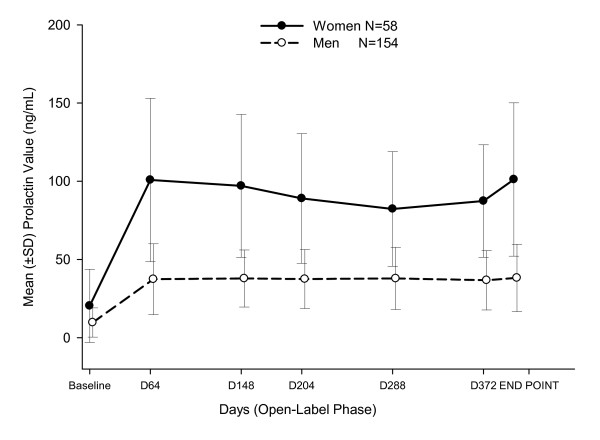
**Mean changes in prolactin overtime**.

Potentially prolactin-related TEAEs occurred in 41 patients (19%; Treatment A [16%; n = 30/186]; Treatment B [42%; n = 11/26]) and led to discontinuation in 3 patients (Treatment A: n = 1; Treatment B: n = 2). The majority of these events were incidental laboratory abnormalities without reported signs and symptoms of clinically relevant hyperprolactinemia. Incidences of potentially prolactin-related TEAEs were higher in women (32.8%, n = 19/58) than in men (14.3%, n = 22/154). Among the clinical signs and symptoms, only libido decreased (n = 4), erectile dysfunction (n = 2) and amenorrhoea (n = 2) were reported as TEAEs in more than 1 patient. Seventy-eight percent of the patients had treatment-emergent abnormal prolactin levels (Treatment A: n = 144 [77%]; Treatment B: n = 21 [81%]). Mean (SD) prolactin levels increased from baseline to endpoint in both women (Treatment A: 78.3 [48.8] ng/mL; Treatment B: 92.3 [45.8] ng/mL) and men (Treatment A: 28.6 [22.00] ng/mL; Treatment B: 29.6 [17.4] ng/mL). Prolactin concentrations were observed to be the highest at the time of C_max _and then decreased to the Day 372 timepoint in women (Figure 5). In men, a steady elevation was present until study endpoint.

Mild TD (described as "increased lip movements") was reported as a TEAE in one patient, which resolved on its own with continued PP treatment. The event was considered as possibly related to study drug. Treatment-emergent adverse events of orthostatic hypotension (n = 18) were mild in severity and did not lead to discontinuation. Treatment-emergent tachycardia (n = 27) and syncope (n = 2) led to discontinuation of 1 patient each in the study. There was no incidence of QT interval corrected (QTc) prolongation > 500 ms in either treatment group, or no patients experienced an increase of > 60 ms from average predose linear-derived QTc (QTc LD) interval. There were no clinically relevant mean changes in laboratory values. Changes in vital sign parameters occurred with 61% of patients experiencing increases in standing pulse rates and 32% experiencing increases in supine pulse rates above clinically important limits (increase ≥ 15 beats/minute and value ≥ 100 beats/minute). Orthostatic changes in blood pressure (defined as a decrease in systolic blood pressure of ≥ 20 mm Hg, or a decrease in diastolic blood pressure of ≥ 10 mm Hg after standing for at least 2 minutes with an increase in pulse rate of ≥ 15 beats per minute) occurred in 22% of patients. Overall, injection-site tolerability was good.

#### Psychiatric symptom evaluations

**Figure 6:**
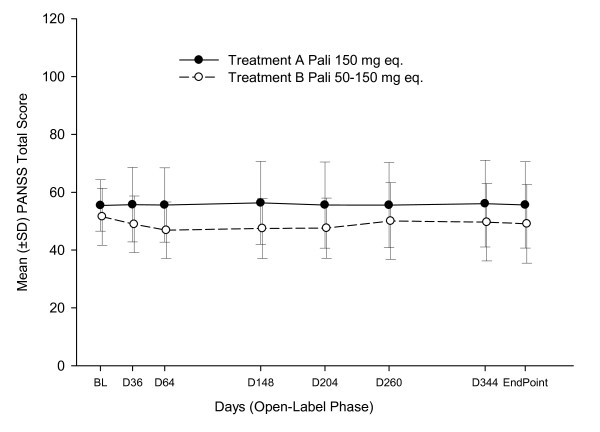
**Mean PANSS total score (+/- SD) over time from baseline-LOCF (Psychiatric Evaluation Analysis Set)**. PANSS- positive and negative syndrome scale; LOCF- Last observation carried forward; Pali - paliperidone palmitate; BL- baseline; D - day.

Of the 212 patients in the safety analysis set, 209 had at least one postbaseline psychiatric evaluation. Patients enrolled in the study were required to be clinically stable with a PANSS score ≤ 70. Mean total PANSS scores remained stable over time (Figure 6). Overall, 30% of all patients enrolled had 30% or greater improvement in the PANSS scores from baseline to endpoint. A total of 204 patients were reported with worsening in PANSS scores from baseline to endpoint, of which 25% of patients had a worsening of 30% or greater. There were no overall clinically meaningful mean changes in either PSP or CGI-S scores in the group over the course of the study, and patients in both treatment groups remained stable.

#### Exploratory analysis outcome

The exploratory analysis showed that the demographic and baseline characteristics were similar among patients who completed the trial while receiving 150 mg eq. throughout and those who did not complete the study at 150 mg eq. dose of PP. Out of 104 patients who completed the study at 150 mg eq. dose of PP throughout, 87 patients (83.7%) experienced at least 1 TEAE; of the 108 patients who did not complete the study at 150 mg eq. dose of PP throughout, 97 (89.8%) reported at least 1 TEAE (Table 5). The TEAEs reported with highest incidences were nasopharyngitis (19.2%, n = 20) and tachycardia (19.2%, n = 20) for those who completed the study at the dose of 150 mg eq.; and insomnia (18.5%, n = 20) for those who did not complete the study at 150 mg eq. dose of PP (Table 5). Five patients (4.8%) of those who completed the study at 150 mg eq. dose of PP; and 28 (25.9%) of those who did not complete the study at 150 mg eq. dose of PP were reported with serious TEAEs (Table 6). Schizophrenia (worsening of symptoms) was the serious TEAE with the highest incidence in those who completed the study at 150 mg eq. dose of PP throughout the study duration (n = 3, 2.9%) as well as in those who did not complete the study at 150 mg eq. dose of PP (n = 8, 7.4%) (Table 6).

**Table 5:** Treatment-emergent adverse events in ≥ 5% of patients based on exploratory analysis of the safety data

	Patients who completed the study on 150 mg eq. throughout			Patients who did not complete the study on 150 mg eq. throughout		
	
	Group A (N = 100)	Group B (N = 4)	Total (N = 104)	Group C (N = 86)	Group D (N = 22)	Total (N = 108)
**Total no. patients with TEAEs**	83 (83.0)	4 (100)	87 (83.7)	76 (88.4)	21 (95.5)	97 (89.8)

Nasopharyngitis	19 (19.0)	1 (25.0)	20 (19.2)	14 (16.3)	3 (13.6)	17 (15.7)

Insomnia	11 (11.0)	1 (25.0)	12 (11.5)	16 (18.6)	4 (18.2)	20 (18.5)

Weight increased	10 (10.0)	1 (25.0)	11 (10.6)	4 (4.7)	4 (18.2)	8 (7.4)

Upper respiratory tract infection	6 (6.0)	1 (25.0)	7 (6.7)	3 (3.5)	2 (9.1)	5 (4.6)

Injection site pain	15 (15.0)	0	15 (14.4)	10 (11.6)	7 (31.8)	17 (15.7)

Headache	13 (13.0)	0	13 (12.5)	11 (12.8)	4 (18.2)	15 (13.9)

Blood prolactin increased	7 (7.0)	0	7 (6.7)	8 (9.3)	4 (18.2)	12 (11.1)

Tachycardia	20 (20.0)	0	20 (19.2)	4 (4.7)	3 (13.6)	7 (6.5)

Orthostatic hypotension	9 (9.0)	0	9 (8.7)	6 (7.0)	3 (13.6)	9 (8.3)

Anxiety	7 (7.0)	0	7 (6.7)	4 (4.7)	4 (18.2)	8 (7.4)

Diarrhoea	6 (6.0)	0	6 (5.8)	6 (7.0)	1 (4.5)	7 (6.5)

Hypertension	8 (8.0)	0	8 (7.7)	1 (1.2)	2 (9.1)	3 (2.8)

Tremor	6 (6.0)	0	6 (5.8)	0	0	0

Pyrexia	5 (5.0)	1 (25.0)	6 (5.8)	0	0	0

Toothache	6 (6.0)	0	6 (5.8)	0	0	0

Akathisia	0	0	0	9 (10.5)	6 (27.3)	15 (13.9)

Psychotic disorder	0	0	0	8 (9.3)	1 (4.5)	9 (8.3)

Schizophrenia	0	0	0	8 (9.3)	1 (4.5)	9 (8.3)

Hyperprolactinaemia	0	0	0	5 (5.8)	3 (13.6)	8 (7.4)

Agitation	0	0	0	5 (5.8)	2 (9.1)	7 (6.5)

Note: Group A consists of all patients who completed the study on 150 mg eq. and stayed on intensive PK sampling throughout; and Group B consists of all patients who completed the study on 150 mg eq. but were not on intensive PK sampling. Group C consists of patients who withdrew early while on 150 mg eq and intensive PK sampling. Group D consists of patients who completed the trial while on flexible dose and switched to non-intensive PK sampling (N = 9); patients who withdrew from the trial while on 150 mg eq. and switched to non-intensive sampling (N = 3); and patients who withdrew from the trial while on flexible dose and switched to non-intensive sampling (N = 10)

**Table 6:** Treatment-emergent serious adverse events based on exploratory analysis of the safety data

	Patients who completed the study on 150 mg eq. throughout			Patients who did not complete the study on 150 mg eq. throughout		
	
	Group A (N = 100)	Group B (N = 4)	Total (N = 104)	Group C (N = 86)	Group D (N = 22)	Total (N = 108)
**Total no. patients with serious TEAEs**	5 (5.0)	0	5 (4.8)	24 (27.9)	4 (18.2)	28 (25.9)

Schizophrenia	3 (3.0)	0	3 (2.9)	7 (8.1)	1 (4.5)	8 (7.4)

Psychotic disorder	1 (1.0)	0	1 (1.0)	5 (5.8)	0	5 (4.6)

Anxiety	1 (1.0)	0	1 (1.0)	1 (1.2)	0	1 (0.9)

Hallucination, auditory	1 (1.0)	0	1 (1.0)	1 (1.2)	0	1 (0.9)

Hallucination, tactile	1 (1.0)	0	1 (1.0)	0	0	0

Agitation	0	0	0	5 (5.8)	0	5 (4.6)

Insomnia	0	0	0	3 (3.5)	0	3 (2.8)

Abnormal behaviour	0	0	0	1 (1.2)	0	1 (0.9)

Aggression	0	0	0	1 (1.2)	0	1 (0.9)

Alcohol abuse	0	0	0	0	1 (4.5)	1 (0.9)

Delusion	0	0	0	0	1 (4.5)	1 (0.9)

Hallucination	0	0	0	1 (1.2)	0	1 (0.9)

Paranoia	0	0	0	1 (1.2)	0	1 (0.9)

Psychiatric decompensation	0	0	0	1 (1.2)	0	1 (0.9)

Schizophrenia, paranoid type	0	0	0	1 (1.2)	0	1 (0.9)

Tremor	0	0	0	2 (2.3)	0	2 (1.9)

Akathisia	0	0	0	1 (1.2)	0	1 (0.9)

Convulsion	0	0	0	1 (1.2)	0	1 (0.9)

Dystonia	0	0	0	0	1 (4.5)	1 (0.9)

Parkinsonism	0	0	0	0	1 (4.5)	1 (0.9)

Salivary hypersecretion	0	0	0	2 (2.3)	0	2 (1.9)

Gastrooesophageal reflux disease	0	0	0	1 (1.2)	0	1 (0.9)

Muscle rigidity	0	0	0	2 (2.3)	0	2 (1.9)

Bradycardia	0	0	0	1 (1.2)	0	1 (0.9)

Irritability	0	0	0	1 (1.2)	0	1 (0.9)

Hypochloraemia	0	0	0	1 (1.2)	0	1 (0.9)

Hyponatraemia	0	0	0	1 (1.2)	0	1 (0.9)

Pneumonia	0	0	0	0	1 (4.5)	1 (0.9)

Note: Group A consists of all patients who completed the study on 150 mg eq. and stayed on intensive PK sampling throughout; and Group B consists of all patients who completed the study on 150 mg but were not on intensive PK sampling. Group C consists of patients who withdrew early while on 150 mg eq. and intensive PK sampling. Group D consists of patients who completed the trial while on flexible dose and switched to non-intensive PK sampling (N = 9); patients who withdrew from the trial while on 150 mg eq. and switched to non-intensive sampling (N = 3); and patients who withdrew from the trial while on flexible dose and switched to non-intensive sampling (N = 10)

EPS-related TEAEs were reported in 16.3% (n = 17) of patients who completed the study at the dose of 150 mg eq. of PP, while an incidence rate of 30.6% (n = 33) was reported in patients who did not complete the study at 150 mg eq. dose of PP. Parkinsonism was the most frequently reported TEAE (n = 7, 6.7%) in patients who completed the study at 150 mg eq. dose of PP; and hyperkinesia was the most frequently reported TEAE (n = 17, 15.7%) in patients who did not complete the study at 150 mg eq. dose of PP.

Prolactin-related TEAEs were reported in 13.5% (n = 14) of patients who completed the study at 150 mg eq. of PP; and 25.0% (n = 27) of those who did not complete the study at 150 mg eq. dose of PP. Increased blood prolactin was the most frequently reported TEAE in both those who completed the study at the dose of 150 mg eq. (n = 12, 11.1%) and those who did not complete the study at 150 mg eq. dose of PP (n = 7, 6.7%).

The mean change [SD] in the body weight and BMI from the open-label baseline to endpoint was high in patients who completed the study at the dose of 150 mg eq. (weight: 4.2 [5.22] kg; BMI: 1.5 [1.74] kg/m^2^) compared with those who did not complete the study at 150 mg eq. dose of PP (weight: 0.6 [5.02] kg; BMI: 0.3 [1.79] kg/m^2^).

## Discussion

This is the first study to examine the long-term pharmacokinetics and tolerability of the highest available dose of PP (150 mg eq.) as fixed dose injections in patients with schizophrenia. Shorter-term fixed-dose studies have shown that PP at doses of 25, 50, 75, 100, or 150 mg eq. was efficacious and tolerable in the treatment of schizophrenia [10,21-24]. Especially as some dose-related effects were observed in these previous studies, it was important to determine if long-term treatment with the maximum available dose was safe and tolerable. In this 1-year phase-1 study of PP in stable patients with schizophrenia, PP was administered either as fixed doses of the highest available 150 mg eq. dose or as flexible doses up to 150 mg eq.). Despite the option for down titration in the flexible-dose group, the majority of patients remained in the fixed dose group, and of the 26 patients opting for flexible dosing, 7 continued on 150 mg eq. dosing. That more than half of the patients completed the trial, and that the majority of those continued on 150 mg eq for the duration of the study attests to the tolerability of the highest available dose during long-term therapy. The results indicate that the safety and tolerability profile of long-term maintenance dosing with PP 150 mg eq. is consistent with that shown in previous short-term studies that included this dose [22,23] as well as with studies across the dose range [10,21,24,25] and no new safety signals emerged. The overall incidence of TEAEs, and the incidences of serious TEAEs, EPS-related and prolactin-related TEAEs were also high in patients who did not complete the study at 150 mg eq. compared with those who completed the study at the dose of 150 mg eq. of PP.

The recommended monthly maintenance dose of PP is 75 mg eq. (117 mg) [9], although some patients may benefit from higher or lower doses.

Generally, the incidence of discontinuations in this long-term study (47%) is consistent with rates observed in previous short duration PP studies [23-25] and also with rates reported in other drug studies in schizophrenia [26,27]. In the current study, patients were exposed to PP 150 mg eq. for a mean duration of 250 days, and approximately half the patients completed the study.

Attainment of optimal therapeutic plasma concentration is important while starting treatment with a LAI [5]. In this study, a median plasma paliperidone concentration of above 7.5 ng/mL, which is associated with a central D2-receptor occupancy of approximately 60% [28], was reached within 2 to 4 days after the first dose. This is within the range (60-80%) associated with antipsychotic efficacy [29-33]. This also confirms that the 150 mg eq. initiation dose administered in the deltoid muscle (using a weight-adjusted needle length) resulted in rapid attainment of target plasma paliperidone concentrations. Steady-state plasma concentrations of paliperidone were reached after approximately 8 to 9 months and the plasma concentrations were within the predicted range by the population PK model. The median concentrations profile of paliperidone during the first 3 months was consistent with those from a previous phase-3 study [23].

The study also allowed an opportunity to look at how the PK results were impacted by ethnicity, given the diversity of demographic characteristics of the study population, with enrollment of a large number of Asian participants. The population PK analyses of this data revealed that, Asian patients had higher plasma exposure to paliperidone than White patients; this could be attributed to the lower BMI in Asians. However, across studies, it has been found that BMI rather than ethnicity, has the most impact on the PK of PP, but this is overcome by the recommended dosing regimen and use of appropriate needle length [34].

Metabolic adverse effects such as dose-related weight gain (≤ 2.6 kg) have been reported in earlier studies with PP [10,21-23]. Consistent with these earlier results, average weight increased (approximately 2.5 kg) in the course of the 1-year study. This weight gain was lower than that observed with other atypical antipsychotics such as olanzapine (4.15 kg over a 10-week treatment period) [35]. Also consistent with previous data, there were a low proportion of treatment-emergent glucose-related adverse events in the current study, and no clinically relevant changes in lipid profile were noted [10,22-24]. Underlying risk factors were identified for each of the patients with glucose-related adverse events, suggesting that PP was not the primary cause.

Approximately one-fourth of the patients experienced at least one EPS-related TEAE during this study. A majority of the EPS-related TEAEs in this study were nonserious. Most of these incidences occurred during the first month of treatment and the rate subsequently decreased to < 5% throughout. There were only 4 reported discontinuations due to EPS-related events. The EPS scales indicated distinct increases in the rates of parkinsonism (16%), while rates for akathisia and dyskinesia were low (1-2%). The proportion of patients requiring anticholinergic medications at endpoint was consistent to those reported earlier for PP [10,21-23]. Despite long-term treatment with the 150 mg eq. dose in this study, only one case of mild TD was reported in this study. The event resolved on continued PP treatment, making the diagnosis of TD uncertain. This low incidence of TD is consistent with results of a previous 33-week study with PP, where only one incidence of TD was reported at 100 mg eq. dose [21]. There were no reports of TD in the previous short-term PP schizophrenia studies across dose ranges 25 to 150 mg eq. [10,22-24].

As expected, increases in prolactin levels were greater in women than in men, consistent with previous data [10,22-24]. However, the incidence of prolactin-related TEAEs was higher in this study than previous phase 3 PP studies, in which the incidence of potentially prolactin-related TEAEs was low (ranging from ≤ 1-2%), consistent with the generally lower doses used and the shorter treatment period of those studies [10,21-24]. The increased prolactin levels in women gradually declined at the end of the study. Importantly, most of the potentially prolactin- related TEAEs observed in this phase-1 study were reported as isolated laboratory abnormalities, and patients were generally asymptomatic with a few exceptions.

There are several limitations to this study. Firstly, this study did not include a placebo group, such that no background rates of TEAE incidences were available for comparison. This limits the clinical interpretation of the data. Also, this study was designed as a safety study and efficacy measurements were secondary objectives. Because the patients enrolled in this study were symptomatically stable, the efficacy results cannot be extrapolated to patients with acute schizophrenia. Though efficacy was not the primary objective of the study, patients generally remained stable both symptomatically and functionally throughout the study. As the average age of patients was between 25-50 years and no elderly patients were included, these results cannot be generalized to either a pediatric or geriatric population. Hyperprolactinemia is a common adverse effect associated with antipsychotic agents. For patients with elevated prolactin concentrations, the practitioners are apt to query for presence of clinical symptoms related to hyperprolactinemia. No specific queries for prolactin-related adverse events were required as per the study protocol. Depending upon the nature of such events and the doctor-patient relationship, patients may or may not be forthcoming in reporting them. Hence, it is possible that prolactin-related adverse events have been underreported.

## Conclusions

Long-term treatment with the highest available dose of PP (150 mg eq.) was tolerable in patients with stable schizophrenia and the pattern of the most common adverse events was generally consistent with the known safety profile for the compound. Injection site tolerability was good. No new safety signals emerged. Paliperidone exposure approached steady-state from the second injection (day 8) onwards, and steady-state levels were reached after 8 to 9 months.

## Abbreviations

PP: Paliperidone palmitate; PK: Pharmacokinetics; SD: Standard deviation; TEAEs: Treatment-emergent adverse events; LAI: Long-acting injectable; PANSS: Positive And Negative Syndrome Scale; BMI: Body mass index; DSM-IV: Diagnostic and Statistical Manual of Mental Disorders: Fourth Edition; LC-MS/MS: Liquid chromatography coupled to tandem mass spectrometry; TD: Tardive dyskinesia; ER: Extended release; EPS: Extrapyramidal symptoms; SAS: Simpson-Angus Rating Scale; AIMS: Abnormal Involuntary Movement Scale; PSP: Personal and Social Performance scale; CGI-S: Clinical Global Impression-Severity scale; C_avg_: Average plasma concentration; QTc: QT interval corrected; QTc LD: Linear-derived QTc interval; LOQ: Limit of quantification; C_predose-_: Plasma concentration measured immediately before the i.m. injections; C_max-_: Maximum plasma concentration; C_min-_: Minimum plasma concentration; t_max_: Time to reach the maximum plasma concentration; AUC_τ-_: Area under the plasma concentration-time curve; C_avg_: Average plasma concentration; FI: Fluctuation Index; PE%: Prediction error percents; |PE|%: Absolute prediction error percents; *PE%_ij -_*: Is the percent prediction error between the *i^th ^*value of the dependent variable in the *j^th ^*subject; and the population prediction of the *i^th ^*observation in the *j^th ^*subject; *DV_ij_*: Is the value of the *i^th ^*observation (i.e. the dependent variable) in the *j^th ^*subject; PRED_ij_: Is the population prediction of the *i^th ^*observation in the *j^th ^*subject.

## Competing interests

AHS was an investigator for this study and has received fees or grants from Janssen Research & Development, LLC., Pfizer, Otsuka, Lundbeck, AstraZeneca, Sanofi Aventis, Servier, Glaxo Smith Kline, Merck Sharp and Dohme, Takeda, Eli Lilly and Dainippon Sumitomo. DC, SG, MS, DH, IN, GP, and YL are employees of Janssen Research & Development, LLC. BR is an employee of Janssen Research & Development, Division of Janssen Pharmaceutica N.V., Beerse, Belgium. DC, SG, MS, DH, IN, GP, BR and YL hold stocks or stock option in Janssen Research & Development, LLC.

## Authors' contributions

All authors met International Council of Medical Journal Editors criteria and all those who fulfilled those criteria are listed as authors. All authors had access to the study data, provided direction and comments on the manuscript, made the final decision about where to publish these data, and approved submission to this journal. Specifically, DC, SG, MNS, DH, IN, AHS, GP, YL and BR contributed to study design, data analysis and interpretation, and literature review. YL oversaw the statistical analyses and provided interpretation of the data. MS and BR oversaw the bioanalyses and provided interpretation of the pharmacokinetic data. All authors read and approved the final manuscript

## Study support

Funded by Johnson & Johnson Pharmaceutical Research & Development, L.L.C, Raritan, N.J., USA (now known as Janssen Research and Development, LLC). The sponsor also provided a formal review of this manuscript. Clinical trial registration no. NCT01150448

## Previous presentations

These data were presented at the 111^th ^Annual meeting of American Society of Clinical Pharmacology and Therapeutics (ASCPT), March 17-20, Atlanta, 2010, American Psychiatric Association (APA) Annual Meeting, May 22-26, New Orleans, 2010, 27^th ^World Congress International College of Neuropsychopharmacology (CINP), June 6-10, Hong Kong, 2010.

## Pre-publication history

The pre-publication history for this paper can be accessed here:

http://www.biomedcentral.com/1471-244X/12/26/prepub

## Supplementary Material

Additional file 1 **PDF, Observed median plasma concentration-time profiles of paliperidone from Asian and White patients**. Click here for file

Additional file 2 **PDF, Distribution of body mass index split by ethnicity. The horizontal lines (and their values) within the boxes represent the medians for the various groups**. The lower and upper edges of the box indicate the 25^th ^and 75^th ^percentiles of the data. The whiskers are the nearest values within 1.5 times the interquartile range below and above the 25^th ^and 75^th ^percentiles, respectively. *represent the outliers. BMI: Body mass index. Click here for file

Additional file 3 **PDF, A.Population pharmacokinetic simulation vs. actual plasma concentration data for White patients B. Population pharmacokinetic simulation vs. actual plasma concentration data for Asian patients**. Click here for file
