# Supplementary material for: A one-year prospective study of the safety, tolerability and pharmacokinetics of the highest available dose of paliperidone palmitate in patients with schizophrenia
Source: BMC Psychiatry. 2012 Mar 28;12:26. doi: 10.1186/1471-244X-12-26 (PMC3384238; doi:10.1186/1471-244X-12-26)

Figure 1. Observed median plasma concentration-time profiles of paliperidone from Asian and White patients

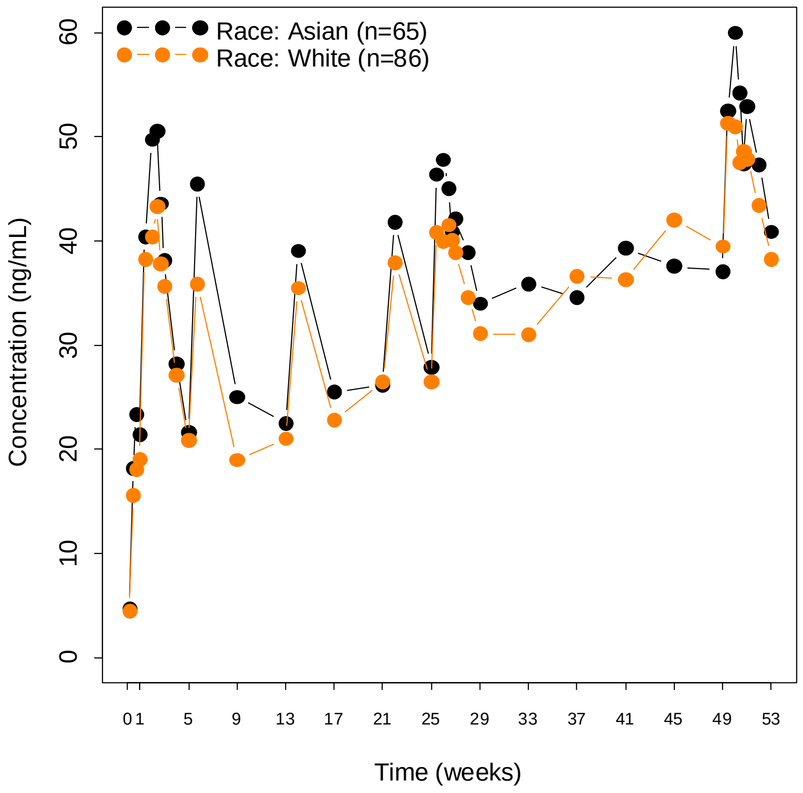

Supplement: Additional file 1 — PDF, Observed median plasma concentration-time profiles of paliperidone from Asian and White patients. [file 1471-244X-12-26-S1.PDF]
