# Supplementary material for: A one-year prospective study of the safety, tolerability and pharmacokinetics of the highest available dose of paliperidone palmitate in patients with schizophrenia
Source: BMC Psychiatry. 2012 Mar 28;12:26. doi: 10.1186/1471-244X-12-26 (PMC3384238; doi:10.1186/1471-244X-12-26)
Supplement: Additional file 2 — PDF, Distribution of body mass index split by ethnicity. The horizontal lines (and their values) within the boxes represent the medians for the various groups. The lower and upper edges of the box indicate the 25th and 75th percentiles of the data. The whiskers are the nearest values within 1.5 times the interquartile range below and above the 25th and 75th percentiles, respectively. *represent the outliers. BMI: Body mass index. [file 1471-244X-12-26-S2.PDF]

Figure 2. Distribution of body mass index split by ethnicity

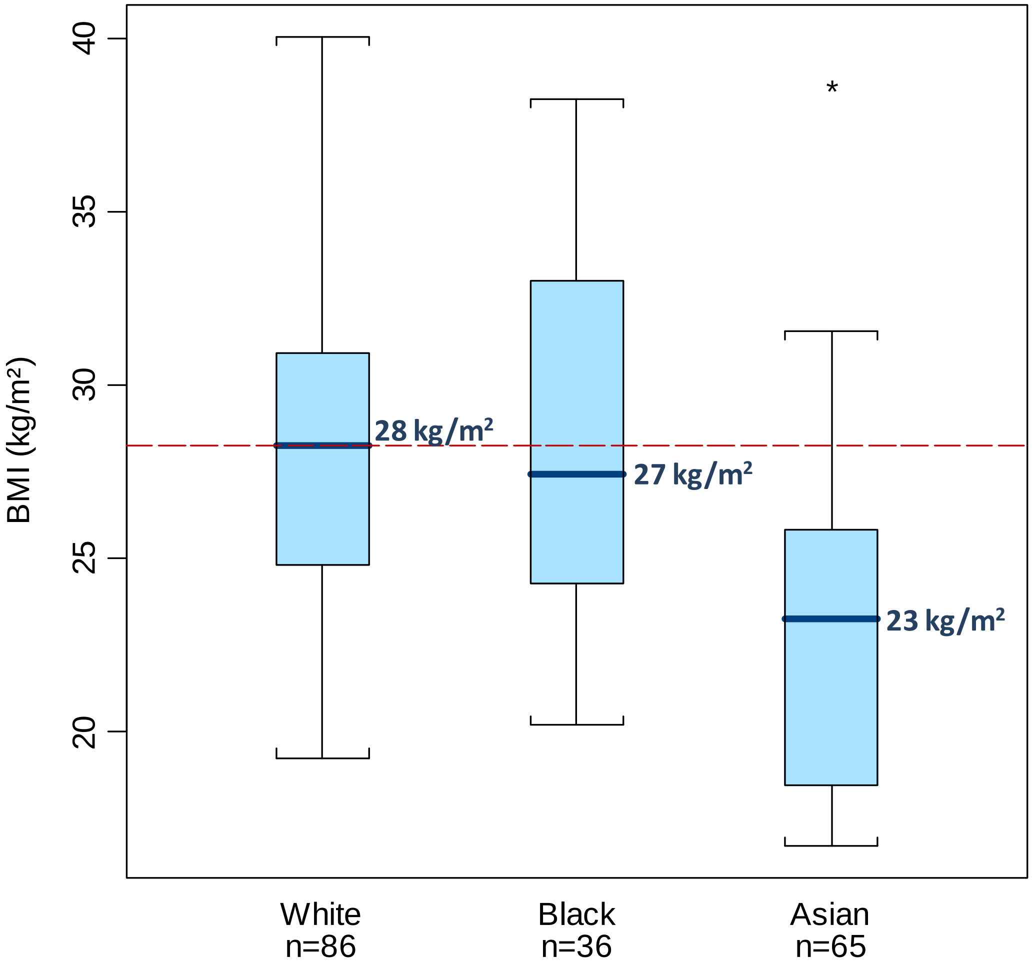

The horizontal lines (and their values) within the boxes represent the medians for the various groups. The lower and upper edges of the box indicate the 25th and 75th percentiles of the data. The whiskers are the nearest values within 1.5 times the interquartile range below and above the 25th and 75th percentiles, respectively.

*represent the outliers.

BMI: Body mass index
