# Supplementary material for: A one-year prospective study of the safety, tolerability and pharmacokinetics of the highest available dose of paliperidone palmitate in patients with schizophrenia
Source: BMC Psychiatry. 2012 Mar 28;12:26. doi: 10.1186/1471-244X-12-26 (PMC3384238; doi:10.1186/1471-244X-12-26)

Figure 3

A. Population pharmacokinetic simulation vs. actual plasma concentration data for White patients

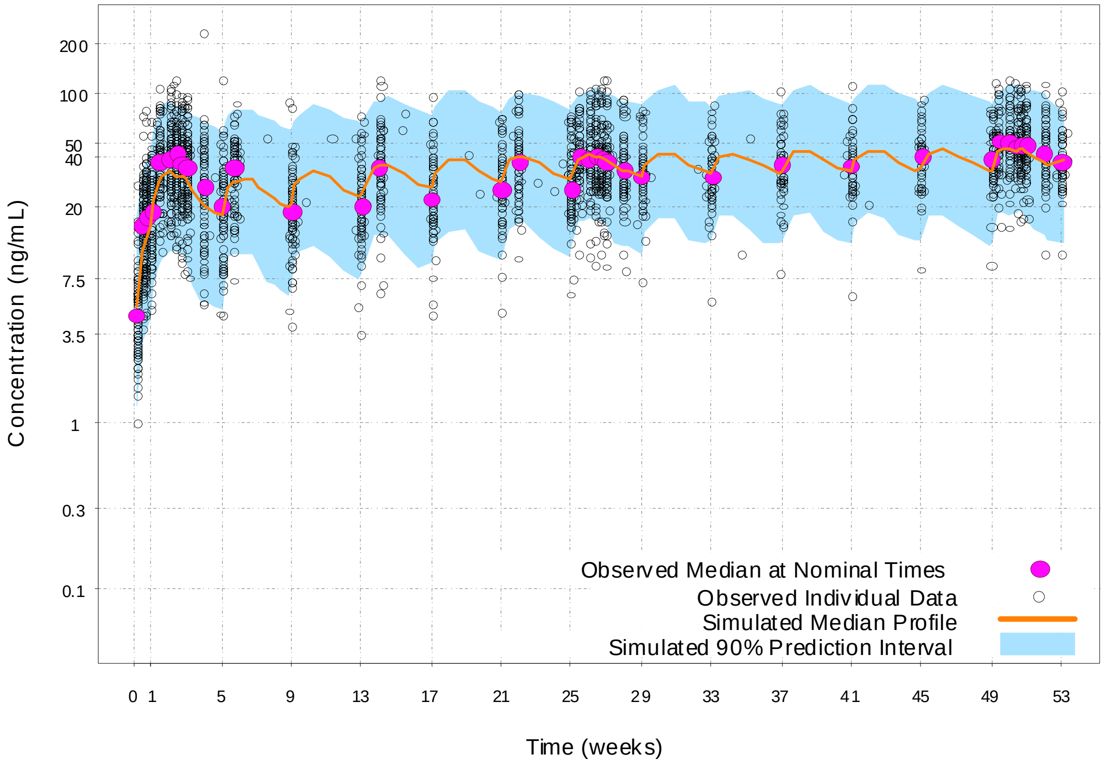

B. Population pharmacokinetics simulation vs. actual plasma concentration data for Asian patients

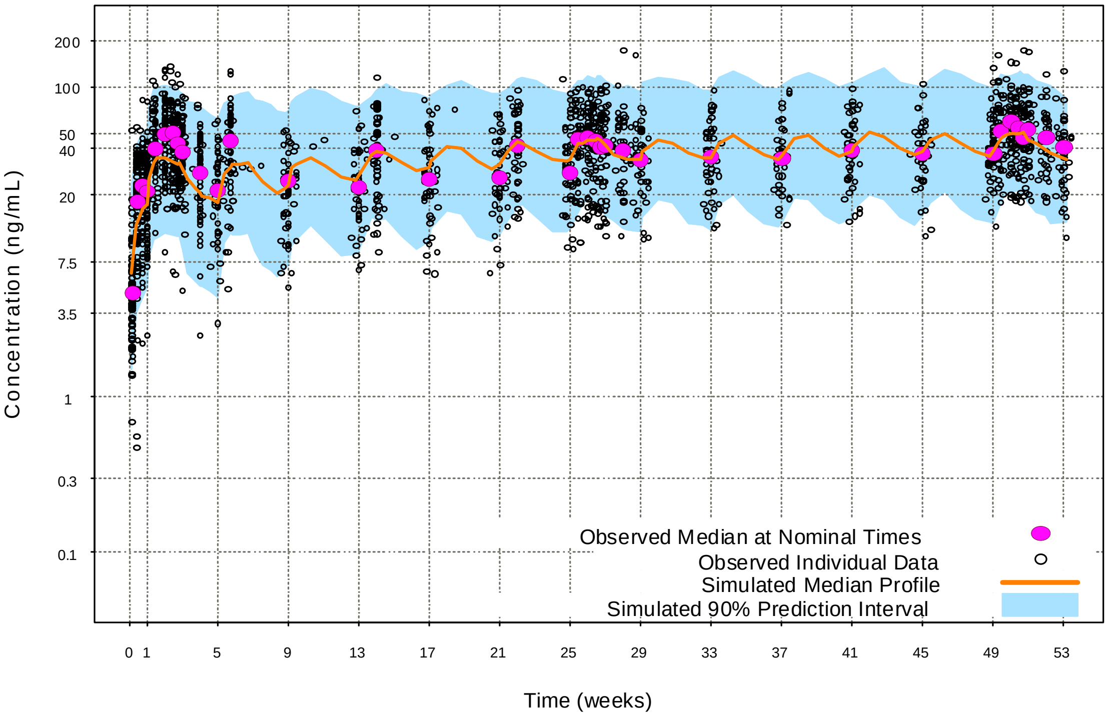

Supplement: Additional file 3 — PDF, A.Population pharmacokinetic simulation vs. actual plasma concentration data for White patients B. Population pharmacokinetic simulation vs. actual plasma concentration data for Asian patients. [file 1471-244X-12-26-S3.PDF]
